# Allelic expression analysis of the osteoarthritis susceptibility locus that maps to chromosome 3p21 reveals *cis*-acting eQTLs at *GNL3* and *SPCS1*

**DOI:** 10.1186/1471-2350-15-53

**Published:** 2014-05-04

**Authors:** Fiona Gee, Clare F Clubbs, Emma VA Raine, Louise N Reynard, John Loughlin

**Affiliations:** 1 Institute of Cellular Medicine, Musculoskeletal Research Group, Newcastle University, Newcastle-upon-Tyne, UK

**Keywords:** Osteoarthritis, Genetic risk, Single nucleotide polymorphism, Allelic imbalance, Linkage disequilibrium

## Abstract

**Background:**

An osteoarthritis (OA) susceptibility locus has been mapped to chromosome 3p21, to a region of high linkage disequilibrium encompassing twelve genes. Six of these genes are expressed in joint tissues and we therefore assessed whether any of the six were subject to *cis*-acting regulatory polymorphisms active in these tissues and which could therefore account for the association signal.

**Methods:**

We measured allelic expression using pyrosequencing assays that can distinguish mRNA output from each allele of a transcript single nucleotide polymorphism. We assessed RNA extracted from the cartilage and other joint tissues of OA patients who had undergone elective joint replacement surgery. A two-tailed Mann–Whitney exact test was used to test the significance of any allelic differences.

**Results:**

*GNL3* and *SPCS1* demonstrated significant allelic expression imbalance (AEI) in OA cartilage (*GNL3*, mean AEI = 1.04, *p* = 0.0002; *SPCS1*, mean AEI = 1.07, *p* < 0.0001). Similar results were observed in other tissues. Expression of the OA-associated allele was lower than that of the non-associated allele for both genes.

**Conclusions:**

*cis*-acting regulatory polymorphisms acting on *GNL3* and *SPCS1* contribute to the OA association signal at chromosome 3p21, and these genes therefore merit further investigation.

## Background

Osteoarthritis (OA) is a common disease of the synovial joint characterised by progressive loss of articular cartilage and often accompanied by changes to the normal function of other tissues of the articulating joint [1]. Epidemiological studies have demonstrated that the disease has a major genetic component, consisting of a large number of susceptibility alleles of small individual magnitude [2]. Recently, several genome-wide association scans (GWAS) have identified susceptibility loci for OA [3-5].

The UK arcOGEN GWAS identified five genome-wide significant loci [3]. The most significant signal, at locus 3p21.1, was associated with hip and knee OA ascertained by joint replacement surgery. This signal was marked by two single nucleotide polymorphisms (SNPs) in perfect linkage disequilibrium (LD; *r*^*2*^ = 1) with one another: rs6976 (C/T) and rs11177 (C/T) (*p* = 7.24 × 10^-11^ and 1.25 × 10^-10^, respectively; odds ratio = 1.12 for the minor alleles). rs6976 is located in the 3-untranslated region (UTR) of *GLT8D1*, which codes for glycosyltransferase 8 domain containing 1, whilst rs11177 is a non-synonymous SNP within exon 3 of *GNL3*, which codes for nucleostemin. The amino acid substitution coded for by rs11177 is benign [3].

Most disease-associated alleles contribute to disease risk by acting as expression quantitative trait loci (eQTLs), influencing the expression or stability of a transcript [6-8]. In OA an excellent example of this is rs143383, which is located in the 5′ untranslated region (UTR) of *GDF5*; the T allele of this SNP correlates with reduced *GDF5* expression in the joint tissues of patients with OA [9]. We hypothesised that the OA association of the 3p21.1 locus may be due to an eQTL acting on one or more genes within the LD block. We therefore assessed allelic expression of genes within the locus in joint tissue from OA patients, using transcript SNPs and pyrosequencing assays that can accurately distinguish the mRNA synthesised from each allele of a gene.

## Methods

### Patients

The Newcastle and North Tyneside research ethics committee granted ethical approval (REC reference number 09/H0906/72) to obtain tissue from patients undergoing elective hip replacement and knee replacement and informed consent was obtained from each donor. Macroscopically normal articular cartilage away from the OA lesion was obtained. Anterior cruciate ligament, fat pad, synovium and meniscus were also obtained from a subset of patients. Additional details regarding the 64 patients studied can be found in Additional file 1. Nucleic acids were extracted from the tissues and cDNA synthesized, as previously described [10,11].

### Transcript SNP selection and genotyping

For *GLT8D1* we used rs6976 whilst for *GNL3* we used rs11177. For each of the other genes studied we chose where possible transcript SNPs that had the highest LD with the association signal (Additional file 2). Genotypes were determined by pyrosequencing.

### Allelic expression analysis

Allelic expression imbalance (AEI) occurs when expression of a gene transcript is higher in the presence of one allele of a SNP than the other. This can occur when the polymorphism alters a transcription factor binding site in a promoter or enhancer region, leading to preferential binding of one or more transcription factors to one transcript compared to the other. We used pyrosequencing to assess AEI for each gene of interest. Pyrosequencing uses a proxy SNP within the gene transcript and entails the quantitative sequencing of the region of DNA containing the SNP to assess the relative amount of transcript produced from each allele [12]. When the proxy SNP is in high or perfect LD with the association SNP, the AEI results can be taken as a direct readout of whether the association SNP correlates with differential expression of the gene of interest. When the proxy SNP is in low LD with the association SNP, the AEI results for the proxy SNP must be stratified by genotype at the association SNP in order to determine whether the association SNP correlates with differential expression of the gene. If this is the case, no AEI will be seen in patients homozygous for the association SNP, whereas AEI in both directions will be observed in heterozygous patients. AEI that does not correlate with the assocation SNP genotype is indicative of an independent eQTL correlating with another SNP and unrelated to the association signal.

Standard PCR was performed to amplify the region of interest and label the product with biotin. Primers used (Sigma-Aldrich, Munich, Germany) are shown in Additional file 3. PCR was performed using the G-Storm GS-4 Q4 Quad Block Thermal Cycler (Somerton Biotechnology Centre, Somerton, UK) under the following cycling conditions: 5 min at 95°C, 38 cycles of 95°C for 30 s, 54–68°C for 30 s, and 72°C for 30 s, followed by 5 min at 72°C. The 20 μl reaction mixture consisted of 25 ng DNA or cDNA, 2 μl 10x PCR buffer, 1–2 mM MgCl_2_, 0.3 μM forward primer, 0.3 μM reverse primer labelled with biotin at the 5′-end, and 0.1U AmpliTaq Gold (Applied Biosystems, Foster City, CA, USA). Samples were analysed on the PyroMark Q24 MDx platform (Qiagen GmbH, Nordrhein-Westfallen, Germany) using the PyroMark Gold Q96 reagents Kit following manufacturer’s instructions. In brief, the reverse primer was labelled with biotin at the 5′-end (Sigma-Aldrich, Munich, Germany) and 20 μl of the amplified DNA/cDNA product was mixed with 1.5 μl Streptavidin Sepharose HP beads (Amersham Biosciences AB, Sweden) and 40 μl binding buffer (10 mM Tris–HCl, pH 7.6, 2 M NaCl, 1 mM EDTA, and 0.1% Tween-20), followed by shaking at 2000 rpm for 10 min. The PCR product-bead complex was captured using a vacuum prep tool, then washed sequentially to remove the non-biotinylated strand with 75% ethanol for 5 s, 0.2 M NaOH for 5 s and washing buffer (10 M Tris acetate, pH 7.6) for 10 s. The beads were released into 25 μl annealing buffer containing 0.3 μM complementary sequencing primer (Sigma-Aldrich) and incubated at 80°C for 2 min. When cooled to room temperature the mixture was loaded into the PyroMark platform with assay-specific software to perform 10 cycles of ATCG dispenses. Sequences were generated automatically and an output of allelic ratio produced using PSQ 96 SQA software (Qiagen).

Samples were analysed in triplicate and only values within one standard deviation from the mean were used. Allelic expression of cDNA was normalised to its corresponding DNA ratio. Accurate discrimination of SNP alleles was verified using artificially created allelic ratios using DNA from homozygous samples.

### Association with OA

We assessed transcript SNPs in *NT5DC2* and *POC1A* for evidence of association to OA using data from the arcOGEN study. This is a large genome-wide association scan conducted in the UK on cases with severe OA of the hip or knee, 80% of whom had undergone total joint replacement, and on population controls [3]. The array used was the Illumina 610 Quad array. Neither SNP is on the 610 Quad array used by arcOGEN, therefore proxy SNPs that are on the array were used. For rs7639267 we used rs10105543 (*r*^*2*^ = 0.902, *D’* = 0.966) whilst for rs747343 we used rs4687805 (*r*^*2*^ = 1, *D’* = 1). Since our expression studies were all performed on OA cases who had undergone total joint replacement, we assessed association using those arcOGEN OA cases who had also undergone this procedure. In total, we studied 5,804 arcOGEN OA cases and 11,009 controls.

## Results

The 3p21.1 association signal contains twelve genes in a 400 kb region [3]. The genes are *STAB1*, *NT5DC2*, *PBRM1*, *GNL3*, *GLT8D1*, *SPCS1*, *NEK4*, *ITIH1*, *ITIH3, ITIH4, MUSTN1* and *TMEM110*. Previous targeted gene expression data by us demonstrated that *NT5DC2*, *PBRM1*, *GNL3*, *GLT8D1, SPCS1* and *TMEM110* were all expressed in cartilage from OA patients, the principal tissue involved in the OA disease process [3 and unpublished observations]. We therefore focused on these genes.

We also wished to determine whether any genes outside of but physically close to the 3p21.1 locus could be subject to eQTLs that correlate with the rs6976 association signal; such genes may be subject to distal-acting *cis* regulation mediated by the signal. We searched the RegulomeDB database for genes known to show differential expression dependent on genotype at rs6976 [13]. This yielded an eQTL at *POC1A* in lymphoblastoid cells. *POC1A* resides telomeric to the association signal and codes for a centrosomal protein. Missense mutations of the gene are involved in the severe osteocutaneous disorder SOFT syndrome, in which growth of bone and ectodermal tissues is arrested leading to a severe form of dwarfism [14]. We demonstrated expression of *POC1A* in OA cartilage (data not shown) and therefore included it with the six cartilage-expressed genes that reside within the signal.

Interrogation of our previously published microarray analysis of cartilage [15] revealed that *GNL3* and *SPCS1* were in the highest quartile of expressed genes, *GLT8D1*, *PBRM1*, *NT5DC2* and *POC1A* fell between the upper and lower quartiles, and *TMEM110* was within the lowest quartile.

### Allelic expression imbalance in cartilage

For each gene we identified individuals heterozygous for the relevant transcript SNP and assayed the relative allelic expression levels in cartilage samples. These samples included tissue from hip and knee joints, and for those SNPs that demonstrated AEI, similar levels of imbalance were detected in both hip and knee samples. Additional file 2 lists the transcript SNPs used and their pairwise *r*^2^ and *D’* values relative to rs6976. If the expression of a gene correlates with the association signal, and if the transcript SNP is in perfect (*r*^2^ = 1) or complete (*D’* = 1) LD with rs6976 we would expect to observe AEI acting in only one direction. If the expression of a gene correlates with the association signal but the transcript SNP is in low LD with rs6976, we would expect AEI to act in both directions, but to correlate with genotype at rs6976.

### *GNL3, SPCS1*, *GLT8D1* and *PBRM1*

**Figure 1:**
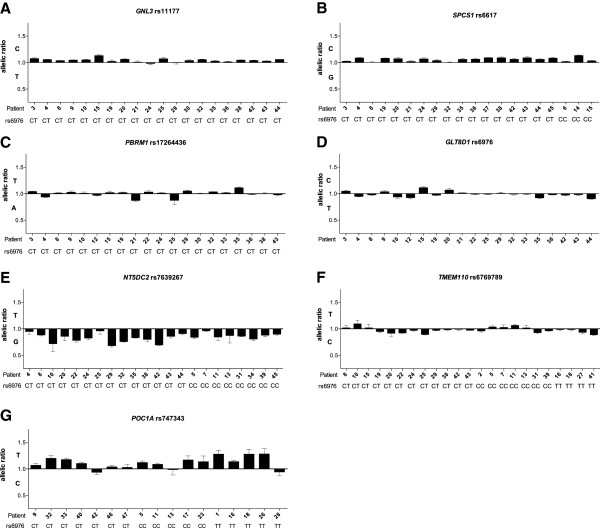
**Allelic expression analysis in cartilage samples from osteoarthritis (OA) patients.** Allelic expression was assessed using the transcript single nucleotide polymorphisms (SNPs) **(A)** rs11177, **(B)** rs6617, **(C)** rs17264436, **(D)** rs6976, **(E)** rs7639267, **(F)** rs6769789 and **(G)** rs747343. Allelic expression of cDNA was normalised to its corresponding DNA. Data is presented as a ratio of expression of the major allele over that of the minor allele; hence a value above 1 means that there is less of the OA-associated allele. Samples are grouped according to the genotype at the OA-associated SNP rs6976, which are shown. Error bars represent the standard error of the mean.

**Figure 2:**
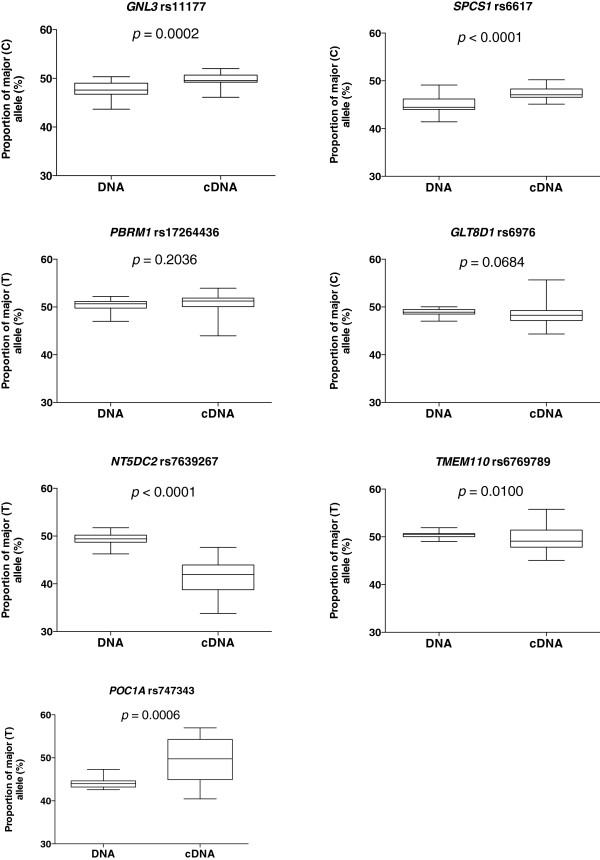
**The proportion of the major allele of each SNP present in DNA and cDNA.** Horizontal lines outside the box represent the smallest and largest value; the horizontal line within the box represents the median value; the limits of the box represent the 25^th^ and 75^th^ percentiles. *p*-values were calculated using a two-tailed Mann–Whitney exact test.

The transcript SNPs for three of these genes are in perfect (*GNL3*) or complete (*SPCS1* and *PBRM1*) LD with rs6976, whilst for *GLT8D1* the transcript SNP is rs6976. For *GNL3* and *SPCS1* we observed AEI in the same direction in the vast majority of patients (Figure 1) and when the data for each gene was combined across all patients (Figure 2) the mean AEI was 1.04 for *GNL3* (*p* = 0.0002) and 1.07 for *SPCS1* (*p* < 0.0001). This data implies that AEI at these two genes correlates with the association signal. For *GLT8D1* and *PBRM1*, AEI was observed in both directions (Figures 1 and 2; *GLT8D1*, mean AEI = 0.99, *p* = 0.0684; *PBRM1*, mean AEI = 1.00, *p* = 0.2036), implying no correlation with the association signal.

### *NT5DC2, TMEM110* and *POC1A*

The transcript SNPs for these three genes are in low LD with rs6976. For *NT5DC2* and *POC1A*, we observed AEI in the same direction for the majority of patients, whilst for *TMEM110* we observed AEI in both directions (Figures 1 and 2; *NT5DC2,* mean AEI = 0.84, *p* < 0.0001; *POC1A*, mean AEI = 1.13, *p* = 0.0006; *TMEM110*, mean AEI = 0.97, *p* = 0.01).

**Figure 3:**
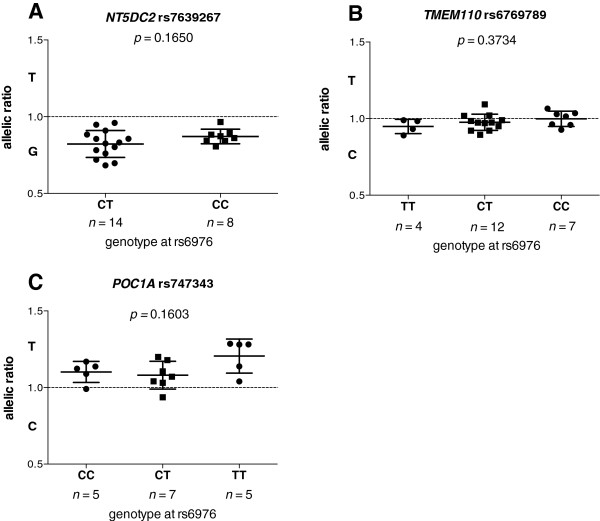
**Columnar scatter plot of allelic ratios in cartilage cDNA stratified by genotype at rs6976.** Allelic ratios of **(A)** rs7639267, **(B)** rs6769789 and **(C)** rs747343 are shown. *n* is the number of patients studied for each group. Error bars in each plot represent the mean and the standard error of the mean. The dashed horizontal lines in each plot show an allelic ratio of 1. *p*-values were calculated using a two-tailed Mann–Whitney exact test for **(A)** and a Kruskal-Wallis test for **(B)** and **(C)**.

For each of these three genes, we plotted the allelic ratios against genotype at rs6976 (Figure 3). No significant correlations were identified (all *p* values > 0.05).

This implies that *NT5DC2* and *POC1A* are subject to eQTLs acting independently of the association SNP rs6976. As the transcript SNPs within *NT5DC2* and *POC1A* appear to be subject to AEI that does not correlate with genotype at rs6976, we assessed whether the eQTLs responsible for the *NT5DC2* and *POC1A* AEIs could also confer an altered risk of OA. We tested the transcript SNPs rs7639267 and rs747343 for association to OA using data previously generated by the arcOGEN GWAS [1]. No associations were observed (both *p* values > 0.05; Additional file 4). Therefore, the *NT5DC2* and *POC1A* eQTLs are unlikely to contribute to OA susceptibility.

### Allelic expression imbalance in other joint tissues

We extended our AEI analysis to include ligament, fat pad, synovium and meniscus samples, all of which were taken from knee joints. All seven genes showed comparable results in these tissues to their cartilage analyses (Additional file 5), implying that the AEI effects observed are active throughout the joint and not restricted to cartilage.

## Discussion

We performed allelic expression analysis of six genes within and one gene close to the 3p21.1 OA association signal. Whilst all seven genes displayed some degree of AEI, our data indicate that only two correlate functionally with OA susceptibility: *GNL3* and *SPCS1*.

*GLT8D1*, *PBRM1* and *TMEM110* demonstrated varying degrees of AEI in most individuals assayed, but there was no correlation between AEI and genotype at rs6976. *NT5DC2* and *POC1A* demonstrated AEI in all individuals assayed and the majority of imbalances operated in the same direction, but again there was no correlation between AEI and genotype at rs6976. The arcOGEN GWAS [3] did not support association between OA and either transcript SNP at *NT5DC2* and *POC1A*. Overall therefore, *cis*-regulation of these five genes does not appear to contribute to the OA association signal marked by rs6976.

*GNL3* and *SPCS1* demonstrated AEI in the same direction in the vast majority of individuals assayed, with the minor allele producing fewer transcripts than the major allele. The fact that not all individuals showed AEI indicates that the AEI at each gene may be modulated by a SNP in high but not perfect LD with rs11177 (*GNL3*) and rs6617 (*SPCS1*). For both genes, the AEI observed in cartilage was small in magnitude but highly significant and was also observed in other joint tissues. As noted in the results, interrogation of our previously published microarray data of cartilage [15] revealed that *GNL3* and *SPCS1* were in the highest quartile of expressed genes. However, in this data set neither gene demonstrated a diffence in expression between OA cartilage and cartilage from non-OA controls. Since AEI directly compares the output from one allele against another it is a more sensitive means of assessing the existence of an eQTL than a microarray analysis. This may account for the absence of an apparent difference in expression of *GNL3* and *SPCS1* between OA and non-OA cartilage in our microarray data.

In this study we have confined our allelic expression analyses to tissue from OA patients. However, we would expect to see similar results if we were to analyse tissue from healthy patients; the allelic expression imbalance observed for *GNL3* and *SPCS1* would be expected to act in all individuals, with the presence of the minor allele of rs6976 leading to lower expression of each gene. However, those individuals in possession of one or more copies of the minor allele of rs6976 would experience an increased risk of developing OA due to lower levels of each gene transcript. A non-OA individual may carry copies of the risk allele but will not develop the disease if they lack risk alleles at other loci; the standard polygenic model.

rs11177 and rs6617 are in high LD (*r*^2^ = 0.932, *D’* = 1), such that the minor alleles for both SNPs are inherited together in a haplotype that correlates tightly with the association signal. Furthermore, the genes are transcribed in the same direction. It appears possible therefore that they are coordinately regulated by a common, polymorphic regulatory element. Functionally, although the association signal correlates with only a small decrease in expression of each gene, the combined effect of lower expression of both genes may contribute to the increased risk of developing OA that maps to this region of the genome.

## Conclusions

In summary, our study reveals that *GNL3* and *SPCS1* are subject to *cis*-acting polymorphisms that influence the expression of the genes in the joint tissues of OA patients, and that this regulation may account for the OA association signal at 3p21.1. *GNL3* codes for nucleostemin, a protein involved in the regulation of the cell cycle and of cell proliferation and self-renewal [16]. It also has a broader role in maintaining nucleolar structure and telomerase activity [17]. OA is characterised by an inability to maintain cartilage tissue and this may be the route through which the reduced expression of *GNL3* is acting to increase OA susceptibility, either on chondrocyte progenitors or on the mature chondrocytes themselves. *SPCS1* codes for signal peptidase complex subunit 1, for which very little is currently known. It is therefore difficult to speculate at this stage as to how reduced expression of *SPCS1* could contribute to OA risk. In order to elucidate how these genes contribute to OA risk a number of molecular investigations could be undertaken. These could include assessment of possible enhancer activity of SNPs in high or perfect LD with rs6976, using a luciferase-based assay in transformed cell lines, and assessment of differential transcription factor binding at such SNPs using electrophoretic mobility shift assays. It would also be informative to assess the effects of *GNL3* and *SPCS1* knockdown on the expression of a panel of genes involved in cartilage maintenance and breakdown, using articular chondrocytes and mesenchymal stem cells undergoing chondrogenesis.

Overall, our results justify detailed investigation of these two genes, and of their encoded proteins, in the context of OA etiology.

## Competing interests

The authors declare that they have no competing interests.

## Authors’ contributions

All authors were involved in drafting the article or revising it critically for important intellectual content, and all authors approved the final version to be published. JL had full access to all the data in the study and takes responsibility for the integrity of the data and the accuracy of the data analysis. Study conception and design: FG, CFC, EVAR, LNR and JL. Acquisition of data: FG and CFC. Analysis and interpretation of data: FG, CFC, LNR and JL.

## Pre-publication history

The pre-publication history for this paper can be accessed here:

http://www.biomedcentral.com/1471-2350/15/53/prepub

## Supplementary Material

Additional file 1 Table of patient characteristics and their genotype at rs6976. Click here for file

Additional file 2 **The seven transcript SNPs and their pair-wise *D’ *** **and *r***^**2 **^**values relative to rs6976.** Click here for file

Additional file 3 Primer sequences used for genotyping and allelic expression analysis. Click here for file

Additional file 4 **Association analyses of transcript SNP proxies in *NT5DC2 *** **and *POC1A *** **with osteoarthritis using arcOGEN data.** Click here for file

Additional file 5 **Allelic expression analysis in joint tissue from osteoarthritis (OA) patients.** Allelic expression was assessed using the transcript single nucleotide polymorphisms (SNPs) **(A)** rs11177, **(B)** rs6617, **(C)** rs17264436, **(D)** rs6976, **(E)** rs7639267, **(F)** rs6769789 and **(G)** rs747343. Allelic expression of cDNA was normalised to its corresponding DNA. Data is presented as a ratio of expression of the major allele over that of the minor allele; hence a value above 1 means that there is less of the OA-associated allele. Samples are grouped according to the genotype at the OA-associated SNP rs6976, which are shown. Error bars represent the standard error of the mean. FP, fat pad; Sy, synovium; Me, meniscus; Li, ligament. Click here for file
