# Supplementary material for: Allelic expression analysis of the osteoarthritis susceptibility locus that maps to chromosome 3p21 reveals cis-acting eQTLs at GNL3 and SPCS1
Source: BMC Med Genet. 2014 May 4;15:53. doi: 10.1186/1471-2350-15-53 (PMC4101866; doi:10.1186/1471-2350-15-53)
Supplement: Additional file 1 — Table of patient characteristics and their genotype at rs6976. [file 1471-2350-15-53-S1.pdf]

Additional file 1. Table of patient characteristics and their genotype at rs6976.

Patient	Sex	Age at surgery (years)	Joint replacement at surgery	Tissue type	rs6976 genotype
1	M	62	K	Cn	TT
2	M	76	K	Cn, FP	CT
3	M	57	K	Cn	CT
4	M	69	K	Cn, Sy	CT
5	F	67	K	Cn	CC
6	F	69	K	Cn	CC
7	F	64	K	Cn	CC
8	M	86	K	Cn	CT
9	F	80	K	Cn	CT
10	F	61	K	Cn	CT
11	M	72	K	Cn	CC
12	M	71	H	Cn	CT
13	M	71	K	Cn	CC
14	F	59	H	Cn	CC
15	M	78	K	Cn	CT
16	M	53	K	Cn	TT
17	M	65	K	Cn	CC
18	M	85	H	Cn	TT
19	F	79	K	Cn	CT
20	F	88	K	Cn	CT
21	F	68	H	Cn	CT
22	F	76	H	Cn	CT
23	F	71	K	Cn	CC
24	F	61	K	Cn	CT
25	F	64	K	Cn	CT
26	F	79	K	Cn	TT
27	F	60	K	Cn	TT
28	M	65	K	Cn	CT
29	F	72	K	Cn	CT
30	M	85	K	Cn	CT
31	F	54	K	Cn	CC
32	F	76	K	Cn	CT
33	F	72	K	Cn	CT
34	F	72	K	Cn	CC
35	F	59	H	Cn	CT
36	M	90	H	Cn	CT
37	F	73	K	Cn	CC
38	F	68	K	Cn	CT
39	M	81	K	Cn	CC
40	M	56	K	Cn	CT
41	F	68	K	Cn	TT
42	F	86	K	Cn	CT
43	M	76	K	Cn	CT

44	M	78	H	Cn	CT
45	F	88	K	Cn	CC
46	F	69	K	Cn	CT
47	M	58	K	Cn	CT
48	F	69	K	Sy	CT
49	F	81	K	FP	CT
50	F	55	K	FP, Sy	CC
51	F	81	K	Sy, Me	CT
52	F	65	K	FP	CC
53	F	78	K	FP, Sy	CT
54	M	76	K	Me	CT
55	M	65	H	Li	CT
56	M	75	K	FP	CT
57	M	74	K	Sy	CT
58	F	78	K	FP, Sy	TT
59	M	56	K	Sy	CT
60	M	67	K	Sy	CT
61	F	67	K	FP	CT
62	F	60	K	FP	CT
63	M	71	K	FP	CT
64	F	73	K	Sy	CT

F, female; M, male; K, knee; H, hip; Cn, cartilage; FP, fat pad; Sy, synovium; Me, meniscus; Li, ligament
