# Supplementary material for: Allelic expression analysis of the osteoarthritis susceptibility locus that maps to chromosome 3p21 reveals cis-acting eQTLs at GNL3 and SPCS1
Source: BMC Med Genet. 2014 May 4;15:53. doi: 10.1186/1471-2350-15-53 (PMC4101866; doi:10.1186/1471-2350-15-53)
Supplement: Additional file 2 — The seven transcript SNPs and their pair-wise D’ and r 2 values relative to rs6976. [file 1471-2350-15-53-S2.pdf]

Additional file 2. The seven transcript SNPs and their pair-wise *D'* and r^2 values relative to the associated SNP rs6976.

**Gene**	**Transcript SNP**	**Alleles (major/minor)**	**MAF**	**Pairwise linkage disequilibrium relative to rs6976**	
				r^2	***D'***
GNL3	rs11177	C/T	0.336	1.000	1.000
SPCS1	rs6617	C/G	0.342	0.932	1.000
PBRM1	rs17264436	T/A	0.317	0.965	1.000
NT5DC2	rs7639267	T/G	0.492	0.521	0.948
TMEM110	rs6769789	T/C	0.336	0.224	0.509
POC1A	rs747343	T/C	0.150	0.038	0.612
GLT8D1	rs6976	C/T	0.336	-	-

MAF, minor allele frequency in Europeans (HapMap CEU)
