# Supplementary material for: Allelic expression analysis of the osteoarthritis susceptibility locus that maps to chromosome 3p21 reveals cis-acting eQTLs at GNL3 and SPCS1
Source: BMC Med Genet. 2014 May 4;15:53. doi: 10.1186/1471-2350-15-53 (PMC4101866; doi:10.1186/1471-2350-15-53)
Supplement: Additional file 3 — Primer sequences used for genotyping and allelic expression analysis. [file 1471-2350-15-53-S3.pdf]

Additional file 3. Primer sequences used for genotyping and allelic expression analysis

SNP	Forward primer (5'-3')	Reverse primer (5'-3')	Sequencing primer (5'-3')
rs6976	CATCTTTTTCCATGGCTTGCTACC	TTCCAGACCCAACAGGCAAATT	GCAACTGTTACTTCCCA
rs11177	GTTCGAGAACATCATCGAAAATTA	TGTTTGGAACTCCTGGGTCTTT	GGCTTCTTGTGACCC
rs6617	TTAGAAGGCCCGGCTACTGA	CGAGGGCGACCGAGACTTA	CGCAGTGCCAGACCT
rs17264436	GTTTCTTCCAGATTACTCA	AGCTGCATATTTTTATGG	CCCCATCTTCATTCAC
rs7639267	CGCATTTCTTCTGCTCAGATC	ACTGCCACACTGTAGGTCTTCAA	GTTGTTTTCTGAGGTGG
rs747343	AGCCGGGGGTTTTCTCTAAAT	ACCCACTTGAGAGGGCTTGT	CACTTGAGAGGGCTTGT
rs6769789	AGCGGGCAGAGACAGCAAG	GCACCGCTTTGGGCTACC	GGTAAGGAACGATGCT
