# Supplementary material for: Allelic expression analysis of the osteoarthritis susceptibility locus that maps to chromosome 3p21 reveals cis-acting eQTLs at GNL3 and SPCS1
Source: BMC Med Genet. 2014 May 4;15:53. doi: 10.1186/1471-2350-15-53 (PMC4101866; doi:10.1186/1471-2350-15-53)
Supplement: Additional file 4 — Association analyses of transcript SNP proxies in NT5DC2 and POC1A with osteoarthritis using arcOGEN data. [file 1471-2350-15-53-S4.pdf]

Additional file 4. Association analyses of transcript SNP proxies in NT5DC2 and POC1A with osteoarthritis (OA) using the arcOGEN data.

SNP	Alleles[1]	MAF in cases	MAF in controls	*p*-value	Odds ratio[2]
rs4687805[3]	A/G	0.1565	0.15	0.1135	1.052 (0.9881-1.119)
rs10105543[4]	G/A	0.3289	0.3189	0.06275	1.047 (0.9976-1.098)

We used the arcOGEN genome-wide association scan dataset, comprising 5,804 OA cases who had undergone hip or knee joint replacement and 11,009 population controls. Neither rs747343 (*POC1A*) or rs7639267 (*NT5DC2*) are on the Illumina 610 Quad array used by arcOGEN. We therefore used the association data generated for SNPs in perfect or complete LD as direct proxies. Stratification of the arcOGEN dataset by sex, by site of OA (hip or knee), and by sex combined with site did not enhance the association *p*-values. MAF, minor allele frequency in Europeans (HapMap CEU). [1]Major/minor. [2](95 % confidence intervals). [3]Proxy SNP for rs747343 (r^2 = 1, D' = 1). [4]Proxy SNP for rs7639267 (r^2 = 0.902, D’ = 0.966).
