# Supplementary figures and images for: Allelic expression analysis of the osteoarthritis susceptibility locus that maps to chromosome 3p21 reveals cis-acting eQTLs at GNL3 and SPCS1
Source: BMC Med Genet. 2014 May 4;15:53. doi: 10.1186/1471-2350-15-53 (PMC4101866; doi:10.1186/1471-2350-15-53)

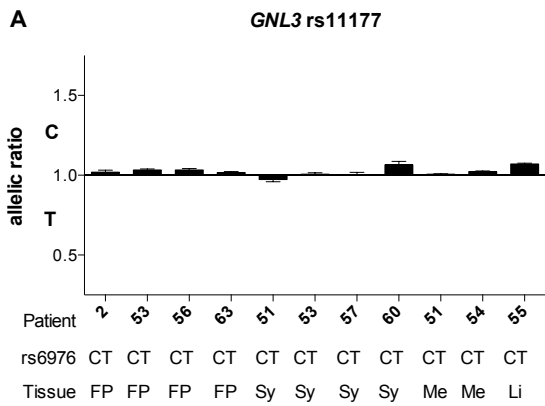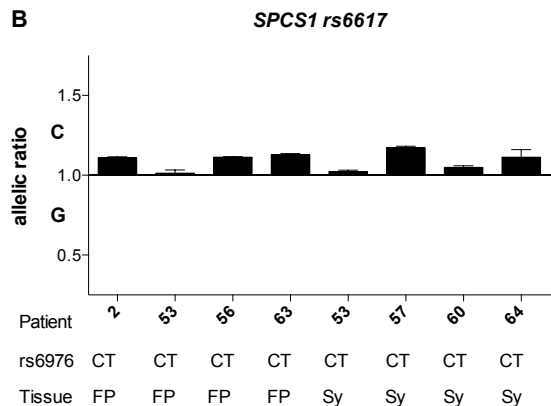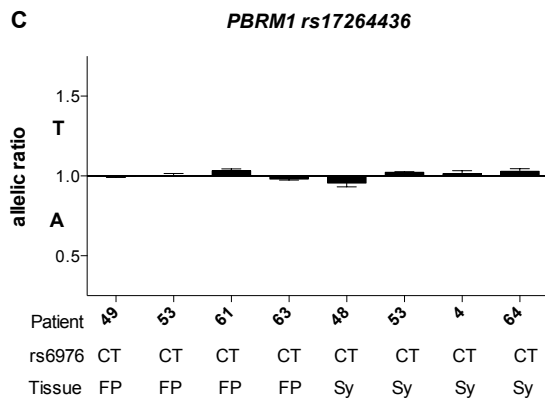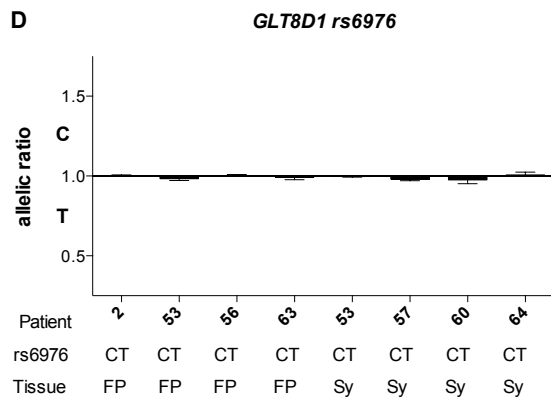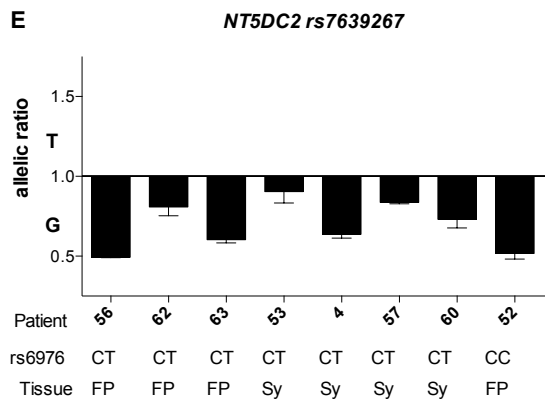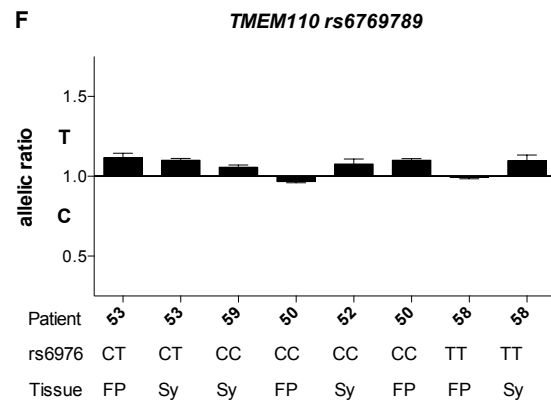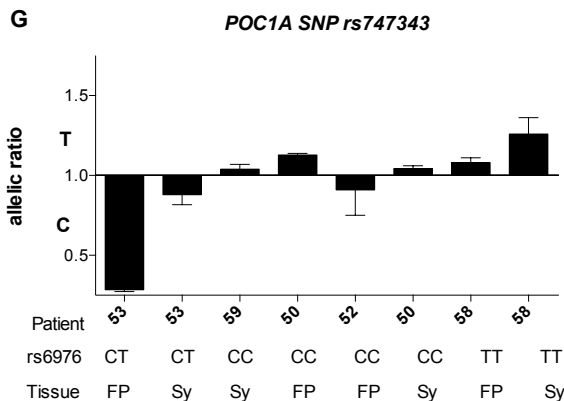

Supplement: Additional file 5 — Allelic expression analysis in joint tissue from osteoarthritis (OA) patients. Allelic expression was assessed using the transcript single nucleotide polymorphisms (SNPs) (A) rs11177, (B) rs6617, (C) rs17264436, (D) rs6976, (E) rs7639267, (F) rs6769789 and (G) rs747343. Allelic expression of cDNA was normalised to its corresponding DNA. Data is presented as a ratio of expression of the major allele over that of the minor allele; hence a value above 1 means that there is less of the OA-associated allele. Samples are grouped according to the genotype at the OA-associated SNP rs6976, which are shown. Error bars represent the standard error of the mean. FP, fat pad; Sy, synovium; Me, meniscus; Li, ligament. [file 1471-2350-15-53-S5.pdf]
